# Supplementary material for: Representation of event and object concepts in ventral anterior temporal lobe and angular gyrus
Source: Cereb Cortex. 2024 Jan 6;34(2):bhad519. doi: 10.1093/cercor/bhad519 (PMC10839851; doi:10.1093/cercor/bhad519)
Supplement: SuppMat_accepted_bhad519 [file suppmat_accepted_bhad519.docx]

**Supplementary Materials**

Supplementary Table 1: Stimuli used in the experiment

| Concept type | Concept | Catch question | Correct Answer |
| --- | --- | --- | --- |
| Event | a ballet | Is it associated with performer | YES |
| Event | a barbeque | Is it associated with suitcase | NO |
| Event | a competition | Is it associated with athlete | YES |
| Event | a concert | Is it associated with bread | NO |
| Event | a date | Is it associated with wall | NO |
| Event | a delivery | Is it associated with customer | YES |
| Event | a diagnosis | Is it associated with doctor | YES |
| Event | a dinner | Is it associated with island | NO |
| Event | an exam | Is it associated with student | YES |
| Event | an exhibition | Is it associated with pill | NO |
| Event | a festival | Is it associated with cactus | NO |
| Event | a fight | Is it associated with boxing | YES |
| Event | a funeral | Is it associated with coffin | YES |
| Event | a graduation | Is it associated with donkey | NO |
| Event | a hike | Is it associated with boots | YES |
| Event | a hunt | Is it associated with oil | NO |
| Event | an interview | Is it associated with reporter | YES |
| Event | a journey | Is it associated with table | NO |
| Event | a lesson | Is it associated with teacher | YES |
| Event | a march | Is it associated with flower | NO |
| Event | a meeting | Is it associated with discussion | YES |
| Event | an opera | Is it associated with tomato | NO |
| Event | an operation | Is it associated with hospital | YES |
| Event | a parade | Is it associated with shelf | NO |
| Event | a party | Is it associated with wine | YES |
| Event | a picnic | Is it associated with rubber | NO |
| Event | a prayer | Is it associated with religion | YES |
| Event | a presentation | Is it associated with package | NO |
| Event | a race | Is it associated with referee | YES |
| Event | a wedding | Is it associated with rat | NO |
| Object | an aeroplane | Is it associated with transport | YES |
| Object | an ant | Is it associated with shark | NO |
| Object | an apple | Is it associated with fruit | YES |
| Object | an arm | Is it associated with lamp | NO |
| Object | a basketball | Is it associated with sports | YES |
| Object | a book | Is it associated with fish | NO |
| Object | a box | Is it associated with water | NO |
| Object | a car | Is it associated with road | YES |
| Object | a castle | Is it associated with brick | YES |
| Object | a child | Is it associated with atom | NO |
| Object | a closet | Is it associated with pants | YES |
| Object | a curtain | Is it associated with tissue | NO |
| Object | a dessert | Is it associated with sugar | YES |
| Object | a dog | Is it associated with star | NO |
| Object | a shirt | Is it associated with clothing | YES |
| Object | a fridge | Is it associated with scissors | NO |
| Object | a hammer | Is it associated with nail | YES |
| Object | a hat | Is it associated with boat | NO |
| Object | a laptop | Is it associated with document | YES |
| Object | a lettuce | Is it associated with police | NO |
| Object | a man | Is it associated with carrot | NO |
| Object | a pan | Is it associated with heat | YES |
| Object | a pencil | Is it associated with eraser | YES |
| Object | a woman | Is it associated with beard | NO |
| Object | a pigeon | Is it associated with bird | YES |
| Object | a steak | Is it associated with roof | NO |
| Object | a toothbrush | Is it associated with bathroom | YES |
| Object | a tree | Is it associated with coral | NO |
| Object | a wheelchair | Is it associated with knife | NO |
| Object | a piano | Is it associated with music | YES |

**
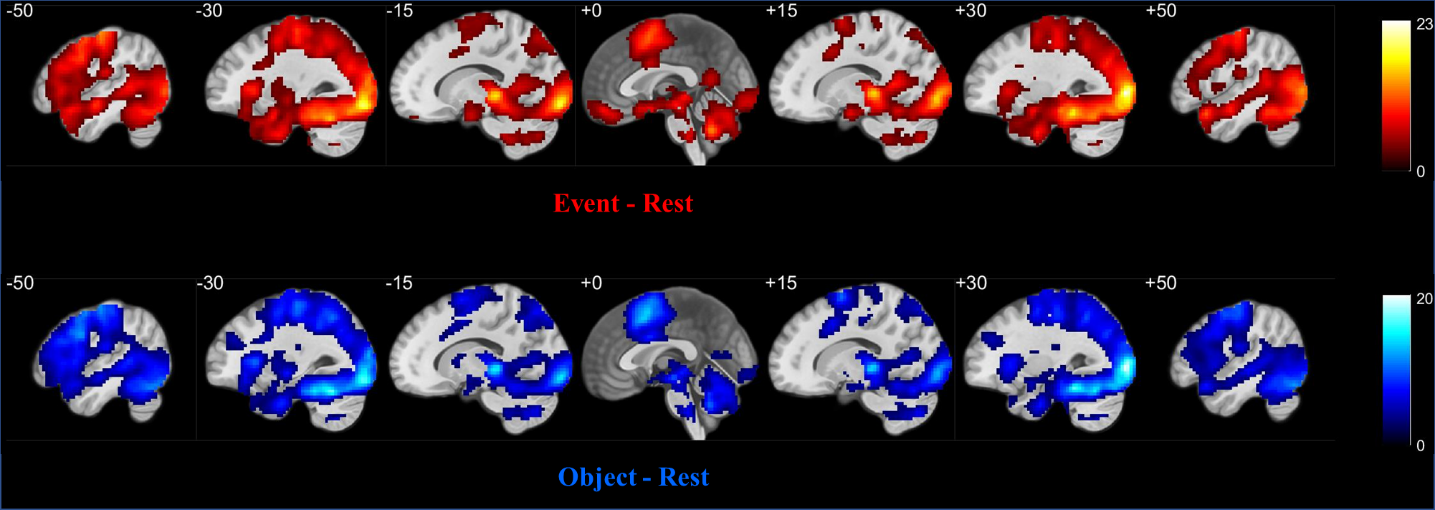
**

*Supplementary Figure 1. Activation for events and objects relative to rest. FWE corrected (p<0.05)*


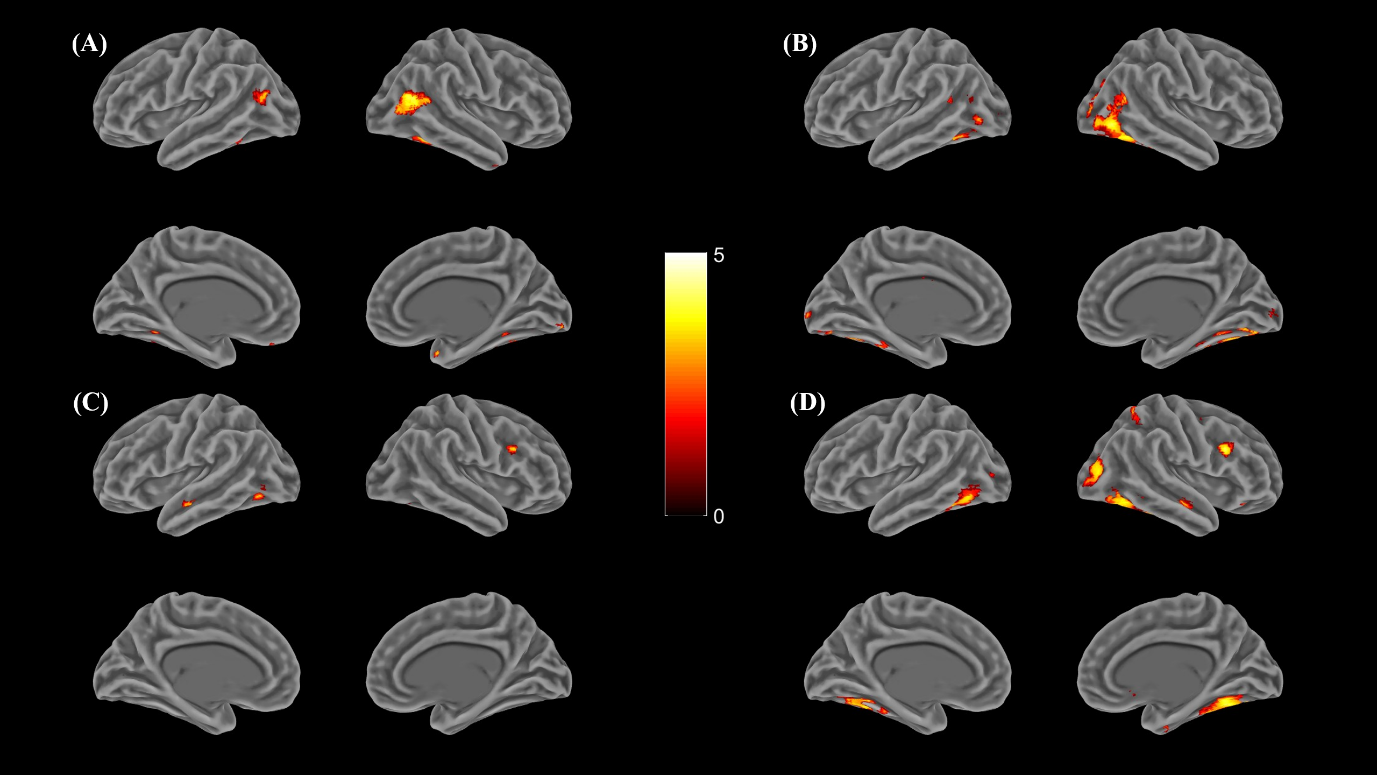


*Supplementary Figure 2. For events > objects, regions showing increased connectivity with (A). Left vATL; (B). Right vATL; (C). Left AG; (D). Right AG. Surface render (p<0.005, no cluster correction)*


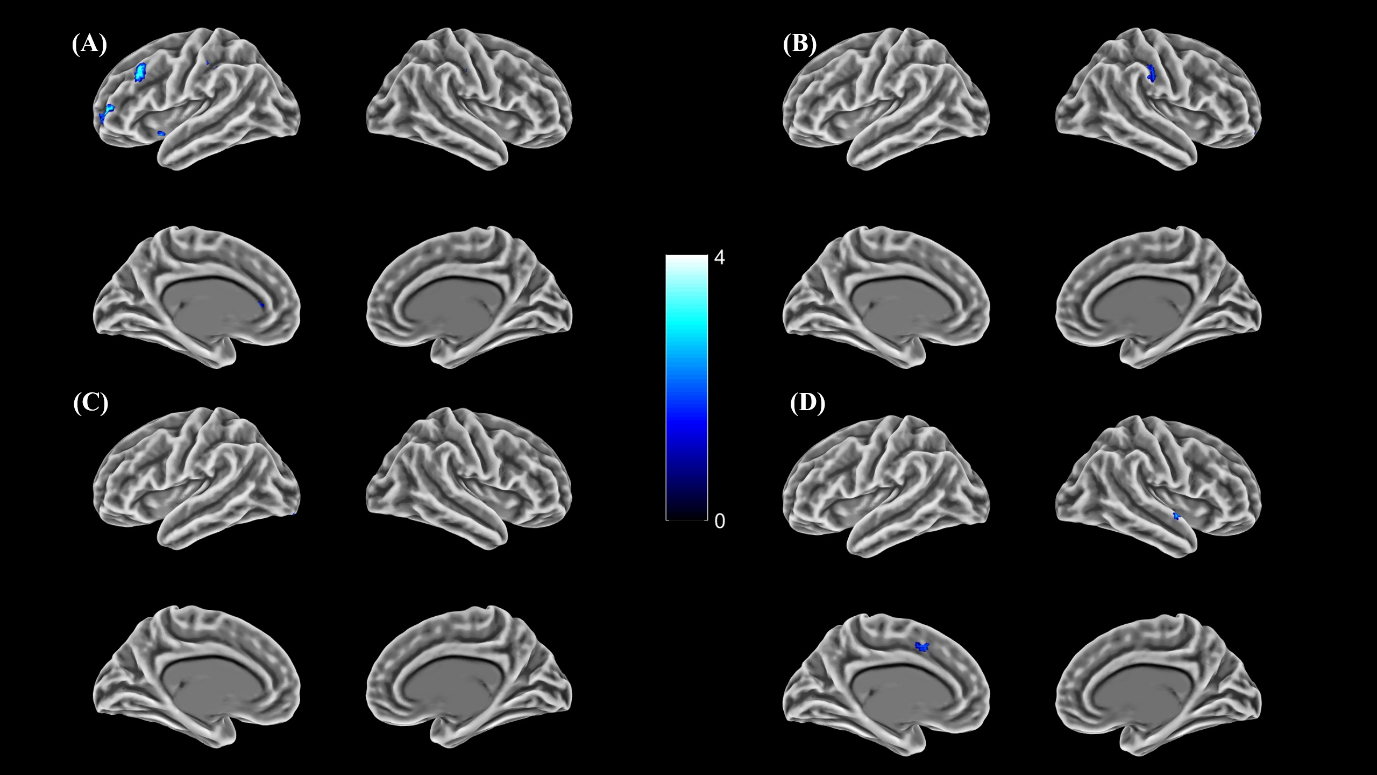


*Supplementary Figure 3. For object > events, regions showing increased connectivity with (A). Left vATL; (B). Right vATL; (C). Left AG; (D). Right AG. Surface render (p<0.005, no cluster correction)*
